# LPS-Induced Intracellular Complement 3 Activation Regulated ATP Production in Yak Rumen Epithelial Cells

**DOI:** 10.3390/vetsci12090841

**Published:** 2025-08-31

**Authors:** Qiang Han, Qiqi Zhang, Duoting Wu, Zihan Yang, Jinyang Huang, Zhisheng Wang, Huawei Zou, Quanhui Peng, Yukun Meng, Yahui Jiang, Jianxin Xiao, Rui Hu

**Affiliations:** 1 Low Carbon Breeding Cattle and Safety Production University Key Laboratory of Sichuan Province, Animal Nutrition Institute, Sichuan Agricultural University, Chengdu 611130, China; 15982183167@163.com (Q.H.); mingrong77@163.com (Q.Z.); duotingwu@163.com (D.W.); shuxiaomu994@163.com (Z.Y.); 17320114607@163.com (J.H.); zswangsicau@126.com (Z.W.); zhwbabarla@126.com (H.Z.); pengquanhui@126.com (Q.P.); xiaojianxin@sicau.edu.cn (J.X.); 2 College of Animal Science and Technology, Sichuan Agricultural University, Chengdu 611130, China; 17838475269@163.com (Y.M.); jiangyahui.ff@163.com (Y.J.)

**Keywords:** yak, rumen epithelial cells, complement 3, ATP, immune inflammation

## Abstract

This study aimed to investigate whether intracellular complement 3 (C3) activation regulates ATP production in yak rumen epithelial cells under inflammatory conditions and its potential mechanism. An in vitro inflammation model was established by stimulating yak rumen epithelial cells with lipopolysaccharide (LPS). Then, protease inhibitors targeting C3 activation enzymes were added. Additionally, to explore the downstream signaling pathway, exogenous C3a and the C3a receptor (C3aR) inhibitor C3aRY were applied to the inflammation model. After treatment with different concentrations of LPS, the gene expression levels and concentrations of pro-inflammatory cytokines, such as *TNF-α*, *IL-1β*, and *IL-6* were significantly up-regulated (*p* < 0.05), while a significant reduction in cellular ATP levels was observed (*p* < 0.05), along with a significant reduction in mitochondrial membrane potential (*p* < 0.05). After treating the inflammation model with different protease inhibitors, the ATP content and gene expression of the ATP synthase subunit *ATP5A* were significantly increased (*p* < 0.05). Exogenous addition of the C3aR inhibitor C3aRY in the inflammation model exhibited a significant increase in ATP content and *ATP5A* gene expression (*p* < 0.05) when compared to the inflammation model. These results demonstrated that intracellular C3 activation inhibited ATP production in yak rumen epithelial cells under inflammatory conditions, likely through C3a–C3aR signaling and the cAMP/PKA pathway.

## Simple Summary

Yaks are an essential source of meat, milk, and livelihood on the Qinghai–Tibet Plateau, yet many young yaks suffer from poor growth and high mortality during the long, cold season. One major reason is inflammation in the rumen, the stomach chamber responsible for nutrient absorption, which reduces the cells’ ability to generate energy. In this study, we examined how a key immune protein called complement 3 (C3) affects energy production in rumen epithelial cells. We created an inflammation model using lipopolysaccharide (LPS), a bacterial component that induces an immune response, and observed that C3 became activated and produced fragments (C3a and C3b) that lowered energy production by disrupting mitochondrial function. When we used specific inhibitors to block C3 activation or prevent its fragment C3a from binding to its receptor (C3aR), the cells produced more energy and released fewer inflammatory molecules. These findings reveal that excessive C3 activation can suppress energy metabolism in rumen cells, while blocking the C3–C3aR pathway may help restore rumen health, improve nutrient use, and support better growth in yaks living under harsh environmental conditions.

## 1. Introduction

Yak (*Bos grunniens*) is the dominant breed of livestock living in the Qinghai–Tibet Plateau (3000 to 5000 m above sea level), which has the characteristics of adaptation to icy conditions and low nutrient intake [1]. From October to May of the following year, the Qinghai–Tibet Plateau has a prolonged cold season with an average temperature ranging from −15 to −5 °C. During the cold season, yaks face a scarcity of forage as the grass withers. Meanwhile, the seasonal reproductive characteristics of yaks result in their pregnancy and neonatal periods predominantly occurring during cold seasons, leading to severe malnutrition that can obstruct their future development and growth [2]. Therefore, the growth-retarded yaks with low body weight, high morbidity, and high mortality significantly reduced the economic benefits of yak farming on the Qinghai–Tibet Plateau [3].

Previous studies have found that rumen epithelium plays an important physiological role in the absorption and transport of nutrients and the protection of the rumen wall [4]. The Volatile Fatty Acids (VFA) are the major energetic substrates for ATP synthesis in the ruminal epithelium [5]. Previous studies have found that excessive production of pro-inflammatory cytokines can alter intracellular cAMP levels, regulate PKA activity, impair mitochondrial function, and thereby reduce ATP production [2,6]. When ATP generation in rumen epithelial cells is impaired, it affects rumen nutrient metabolism and hinders rumen development, making it a key reason for causing growth retardation of yaks [7]. Furthermore, existing studies have shown that the activation of complement component C3 (C3) can regulate mitochondrial respiration and glucose utilization in cells [8], suggesting that inflammation and C3 activation are closely related to cellular energy metabolism.

C3 is the most abundant component in serum and plays a central role in innate immunity [9,10]. Unlike the extracellular activation of C3 by convertases, intracellular C3 is stored in lysosomes and endosomes of T cells and can be activated by cathepsins to generate C3a and C3b [11]. C3a and its receptor C3aR regulate mitochondrial biogenesis, oxidative phosphorylation, and ATP production via cAMP/PKA signaling, while also modulating inflammatory responses [12,13]. CD46, the receptor of C3b, further participates in T-cell and other immune cells activation [14]. Notably, the upstream triggers of C3 activation appear to be cell-type specific: in immune cells, it is largely associated with pathogen recognition, whereas in non-immune cells, such as adipocytes and intestinal epithelial cells, it can be induced by stress signals including LPS or metabolic imbalance [15].

Most studies on the mechanism of C3 intracellular activation that regulates cellular metabolism mainly focus on the immune cells of humans and mice. Rumen epithelium is a stratified squamous epithelium, which is different from the simple columnar epithelium, such as intestinal epithelium. Therefore, it was unclear whether intracellular activation of C3 occurred in rumen epithelial cells induced by LPS. It is also unclear how intracellular activation of C3 regulates the mitochondrial ATP production in rumen epithelial cells under an inflammatory state. In this study, LPS was added exogenously to establish an inflammatory yak rumen epithelial cell model, to study the mechanism of intracellular activation of C3 and its regulation on the ATP production in yak rumen epithelial cells.

## 2. Materials and Methods

### 2.1. Source of Rumen Epithelial Cells and Ethics Statement

Yak rumen epithelial cells were selected from the immortalized yak rumen epithelial cell line, from a previously established cell line derived from yak rumen tissue [16]. The experimental protocol used in the present study was approved by the Animal Policy and Welfare Committee of the Agricultural Research Organization of Sichuan Province, China, and was in accordance with the guidelines of the Animal Care and Ethical Committee of Sichuan Agricultural University (#SCAUA2021-36).

### 2.2. Resuscitation and Cultivation of Rumen Epithelial Cells in Yak

The frozen immortalized yak rumen epithelial cell line was quickly thawed in a 37 °C water bath. Centrifuge at 1000 r/min for 3 min and discard the supernatant. Add 1 mL of complete medium (89% 1640 medium + 1% triple antibiotics + 10% fetal bovine serum), resuspend, and then seed in a 25 cm^2^ culture flask. Incubate at 37 °C with 5% CO_2_ until the cell density reaches over 90%. Then digest and pass the cells.

### 2.3. Experimental Design

To establish an inflammatory model of rumen epithelial cells, the yak rumen epithelial cells, after digestion and centrifugation, were inoculated into 6-well plates according to the appropriate density. When the cells were fused to 80~90%, the cells were treated with LPS from *Escherichia coli* O111:B4 (Sigma-Aldrich, St Louis, MS, USA) to induce an inflammatory response in yak rumen epithelial cells of 0, 10, 20, 40, 80, 160 μg/mL for 24 h [17], respectively, and each processing setting has 6 repetitions to establish an inflammation model.

To investigate the effects of intracellular activation of C3 on ATP production in the rumen epithelial cells, rumen epithelial cell was divided into 5 groups, including blank groups, LPS group, LPS + CA-074ME1 group, LPS + Cathepsin L Inhibitor I group, and LPS + Cathepsin Inhibitor 1 group. The blank group was treated with the complete medium as a control group. The LPS group was treated with additional LPS in the complete medium. Based on the LPS group, the LPS + CA-074ME1 group was treated with 20 nmol/L CA-074ME (Santa Cruz, CA, USA), which is the inhibitor of cathepsin B, a potential cathepsin to stimulate C3 intracellular activation. The LPS + Cathepsin L Inhibitor I group was treated with 10 nmol/L cathepsin L Inhibitor I (Santa Cruz, CA, USA), which is the inhibitor of Cathepsin L, another potential cathepsin to stimulate C3 intracellular activation. The LPS + Cathepsin Inhibitor 1 group was treated with 10 nmol/L cathepsin Inhibitor 1 (Santa Cruz, CA, USA), which is a pan-inhibitor for cathepsins in the cells. The appropriate concentration of each inhibitor was optimal in the pre-experiment. After the cells were fused to 80~90%, the different cathepsin inhibitors were added to pretreat the cells for 2 h, and then LPS was added to treat the cells for 24 h [18].

Additionally, to explore the impact of intracellular C3 downstream activation on ATP production in yak rumen epithelial cells, the rumen epithelial cells were divided into 4 groups, including blank groups, LPS group, LPS + C3a group, and LPS + C3aRY group. The blank group was treated with the complete medium as a control group. The LPS group was treated with additional LPS in the complete medium. Based on the LPS group, the LPS + C3a group was treated with 1 μmol/L C3a protein (Monmouth Junction, NJ, USA). The LPS + C3aR group was treated with 30 nmol/L C3aRY (Monmouth Junction, NJ, USA), which is a C3a receptor antagonist inhibitor. The appropriate concentration of protein inhibitor was selected according to both pre-experiments in our laboratory and previous studies that applied comparable ranges in cell models [19,20]. After the cells were fused to 80~90%, the protein and inhibitor were added to pretreat the cells for 1 h, and then LPS was added to treat the cells for 24 h [21].

### 2.4. Cell Viability Assay

Cell viability was evaluated using the Cell Counting Kit-8 (CCK-8; Dojindo Laboratories, Kumamoto, Japan) according to the manufacturer’s instructions. Yak rumen epithelial cells were seeded in 96-well plates at a density of 1 × 10^4^ cells per well and incubated overnight at 37 °C with 5% CO_2_. Cells were then treated with various concentrations of LPS for 24 h. After treatment, 10 μL of CCK-8 solution was added to each well, followed by incubation for 2 h. The absorbance at 450 nm was measured using a microplate reader (BioTek Instruments, Winooski, VT, USA). Cell viability was calculated as a percentage of the absorbance value of the untreated control group.

### 2.5. Determination of Inflammatory Cytokine and C3 Level

The adherent cells were gently washed with 1× cold phosphate-buffered saline (PBS; Solarbio, Beijing, China), then digested with a 0.25% trypsin solution (Solarbio, Beijing, China), and centrifuged at 1000× *g* for 5 min. Suspension cells can be collected directly by centrifugation. The collected cells were washed 3 times with cold PBS. 150–200 μL PBS was added to every 1 × 10^6^ cells and the cells were broken by repeated freezing and thawing (the volume of PBS could be reduced if the content was very low). The extraction solution was centrifuged at 1500× *g* for 10 min, and the concentrations of pro-inflammatory factors Interleukin-6 (IL-6), interleukin-1β (IL-1β), tumor necrosis factor-α (TNF-α), C3a and C3b were detected in the supernatant by ELISA kit, with six replicates per group (Ruixinbio, Quanzhou, China; IL-6 kit no.RX1600853B, IL-1β kit no.RX1600856B, TNF-α kit no.RX1600738B, C3a kit no.RX1600916B, C3b kit no.RX1600223B) [22].

### 2.6. RNA Extraction and qRT–PCR Analysis of Gene Expression

Total RNA extraction kit (Yeasen Biotechnology, Shanghai, China) was used to extract total RNA from cells. Gene expression was determined by qPCR as previously reported [23]. An OD 260/280 value greater than 1.8 was required. The Hifair™ II 1st Strand cDNA Synthesis Super Mix for qPCR kit was used (Yeasen Biotechnology, Shanghai, China) for reverse transcription. Reverse transcription was not less than 1 μg total RNA in a 20 μL reaction volume. The Hieff UNICON^®^ Universal Blue qPCR SYBR Green Master Mix kit was used (Yeasen Biotechnology, Shanghai, China) for qPCR. PCR was performed using SYBR Green real-time PCR master mix in a qPCR system (QuantStudio 5, Applied Biosystems, Waltham, MA, USA). qPCR cycles consisted of 95 °C for 2 min, followed by 40 cycles of 95 °C for 10 s and 60 °C for 30 s. β-Actin was used as the internal control. The PCR primers used in the quantitative assays are listed in Supplementary Table S1. The fold change in mRNA expression was determined using the 2^−ΔΔCt^ method.

### 2.7. Detection of ATP Concentration

The cells were collected in the centrifuge tube, and the supernatant was discarded. 1 mL extract was added into 5 million cells, then crushed by ultrasonic wave (Ice bath, power 200 W, ultrasound 2 s, stop 1 s, total time 1 min), and centrifuged at 10,000× *g* 4 °C for 10 min; Take the supernatant into another EP tube, add 500 mL chloroform to shake and mix thoroughly, centrifuge at 10,000× *g* 4 °C for 3 min. The supernatant was collected and kept on ice for ATP concentration measurement using an ATP assay kit (BC0305, Solarbio Science & Technology, Beijing, China) according to the manufacturer’s instructions [24], Luminescence was measured using a microplate reader (Thermo Scientific Varioskan LUX, Thermo Fisher Scientific, Waltham, MA, USA), and ATP concentrations were calculated based on a standard curve generated from known ATP standards.

### 2.8. Mitochondrial Membrane Potential Monitored by JC-1

The 10^5^ to 6 × 10^5^ cells were taken and re-suspended in 0.5 mL cell culture medium, which could contain serum and phenol red. Add 0.5 mL JC-1 dyeing solution, reverse several times, and mix well. The cells were incubated at 37 °C for 20 min. After incubation at 37 °C, centrifuge at 600× *g* 4 °C for 3~4 min and precipitate cells. Discard the supernatant and wash with JC-1 staining buffer (1×) (Solarbio, Beijing, China) twice: add 1 mL JC-1 staining buffer (1×) to suspend the cells, centrifuge at 600× *g* 4 °C for 3~4 min, precipitate the cells, and discard the supernatant. Then 1 mL JC-1 staining buffer (1×) was added to the suspended cells and centrifuged at 600× *g* 4 °C for 3~4 min, the cells were precipitated, and the supernate was discarded. The appropriate amount of JC-1 staining buffer (1×) was re-suspended and analyzed by flow cytometry [25].

### 2.9. Detection of Organic Acids

The culture medium in the culture plate was removed and cleaned with PBS. The cells were collected into a 1.5 mL centrifuge tube and centrifuged at 4000 r/min for 3 min. After the supernatant was sucked out, 0.2 mL 2.5% perchloric acid was added into the centrifuge tube and ultrasound for 1 h. 13,000 r/min centrifugation for 30 min. The concentration of Malic acid (MA), Citric Acid (CA), Pyruvic acid (PA), and Lactic acid (LA) (Sigma, St. Louis, MO, USA)was determined by high-performance liquid chromatography(HPLC) [26]. HPLC was coupled with a variable wavelength UV detector, and a reverse-phase (RP) Agilent XDB-C18 column (250 mm × 4.6 mm I.D., 5 μm particle size) was used. All procedures were carried out using 4.5% metaphosphoric acid (pH 2.20), filtered through a 0.45-µm membrane (0.45 µm, AFO-0504, Millipore, Billerica, MA, USA), as the eluent at a flow rate of 0.7 mL/min. This mobile phase must be prepared fresh daily. The column was a Spherisorb ODS-2 S5 (250 mm × 4.6 mm I.D., particle size 5 μm) thermostated at 25 °C. The injection volume was 20 µL and all standards were injected in triplicate. The optimum wavelength for the simultaneous determination of the organic acids was 215 nm.

### 2.10. Statistical Analysis

All data were represented as mean and standard error. Statistical analyses were performed using SPSS 27.0, including normality testing, one-way analysis of variance (ANOVA), and Duncan’s multiple range test for post hoc comparisons. Experimental data were visualized using GraphPad Prism 8.0.0. Differences were considered statistically significant at *p* < 0.05.

## 3. Results

### 3.1. Effects of LPS on the Secretion of Pro-Inflammatory Factors and the Activation of C3 in Rumen Epithelial Cells of Yaks

**Figure 1:**
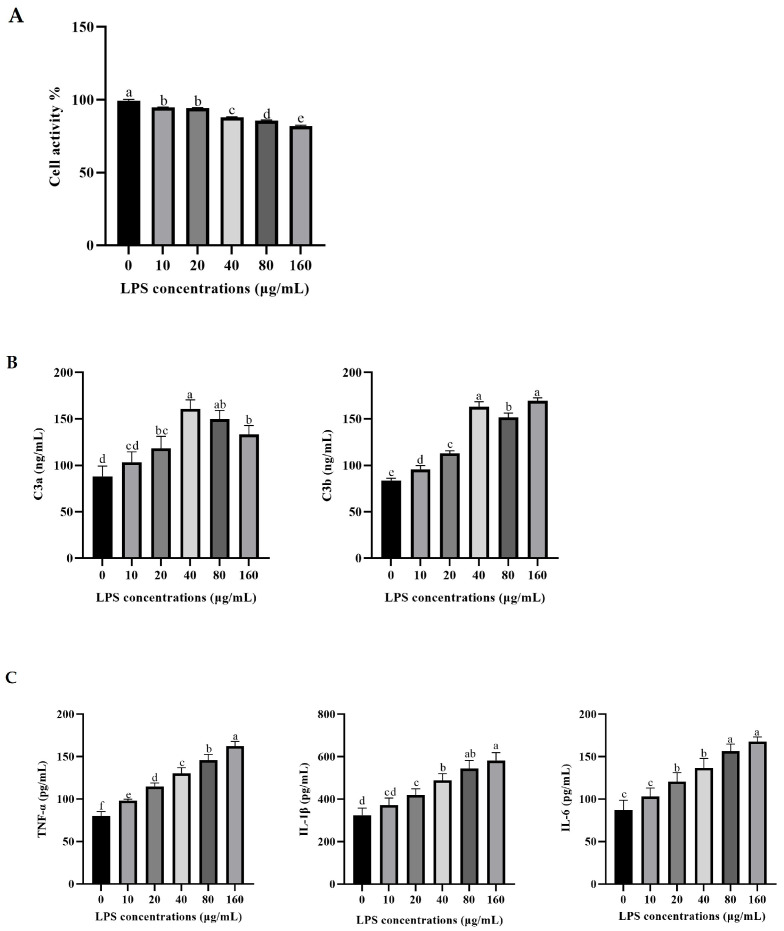
Effects of LPS concentrations on cell viability (**A**), C3 activation products (**B**), and pro-inflammatory cytokine secretion (**C**) in rumen epithelial cells of yaks. The values are presented as mean ± SEM (*n* = 6). Different superscripts (a–f) indicate significant differences (*p* < 0.05).

As the results showed in Figure 1, Compared with the control group, the activity of rumen epithelial cells significantly decreased with increasing concentrations of LPS (*p* < 0.05). In addition, the concentrations of fragments C3a and C3b, the activation products of intracellular C3, increased significantly with the increase in LPS concentration (*p* < 0.05). Meanwhile, the concentrations of pro-inflammatory factors such as TNF-α, IL-1β, and IL-6 in the LPS groups were significantly increased with the LPS concentrations increasing (*p* < 0.05).

### 3.2. Effects of LPS on ATP Production in Rumen Epithelial Cells of Yak

**Figure 2:**
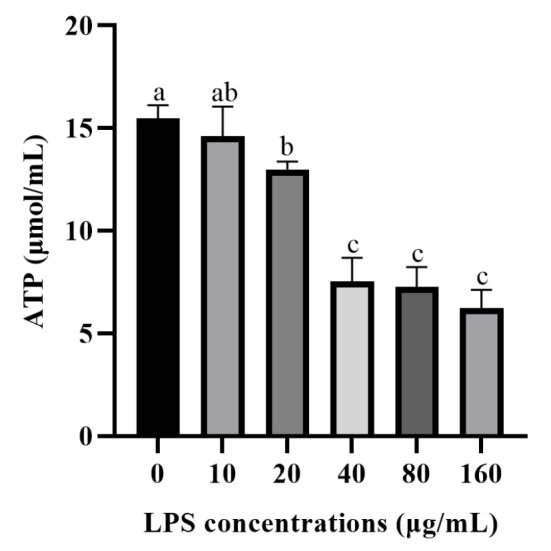
Effects of LPS concentrations on ATP production and in rumen epithelial cells of yak. The values are presented as mean ± SEM (*n* = 6). Different superscripts (a–c) indicate significant differences (*p* < 0.05).

The effect of LPS on ATP production in the rumen epithelial cells of yaks is shown in Figure 2. Compared with the control group, LPS treatment resulted in a decreasing trend of ATP concentration in rumen epithelial cells, and when LPS concentration was 20 μg/mL, ATP content was decreased compared with the control group (*p* < 0.05).

### 3.3. Effects of Inhibited the C3 Activation on Secretions and mRNA Expressions of Pro-Inflammatory Factors of Yak Rumen Epithelial Cells Under an Inflammatory State

**Figure 3:**
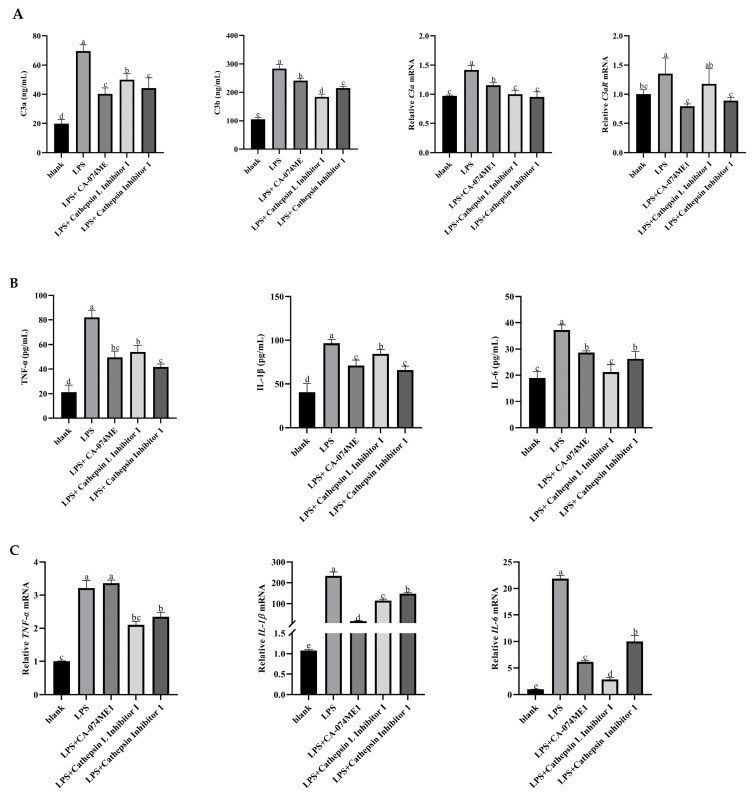
Effects of C3 activation inhibited on C3 activation products (**A**), pro-inflammatory cytokine secretion (**B**), and related gene mRNA expression (**C**) in yak rumen epithelial cells under an inflammatory state. The values are presented as mean ± SEM (*n* = 6). Different superscripts (a–e) indicate significant differences (*p* < 0.05).

Compared with the blank group, the LPS group significantly increased the concentration of fragments C3a and C3b, the gene expression of C3a, and the gene expression of C3aR, the specific receptor of C3a, significantly increased (*p* < 0.05). After the addition of the cathepsin inhibitor, the concentrations of C3a and C3b, the gene expression of C3a, and the gene expression of C3aR were significantly decreased (*p* < 0.05, Figure 3A).

The concentrations and gene expression levels of *TNF-α*, *IL-1β*, and *IL-6* pro-inflammatory factors in the LPS group were significantly increased (*p* < 0.05). However, the concentrations and gene expression levels of these pro-inflammatory factors were significantly decreased after the cathepsin inhibitor was added (*p* < 0.05, Figure 3B,C).

### 3.4. Effects of Inhibited the C3 Activation on ATP Production of Yak Rumen Epithelial Cells Under an Inflammatory State

**Figure 4:**
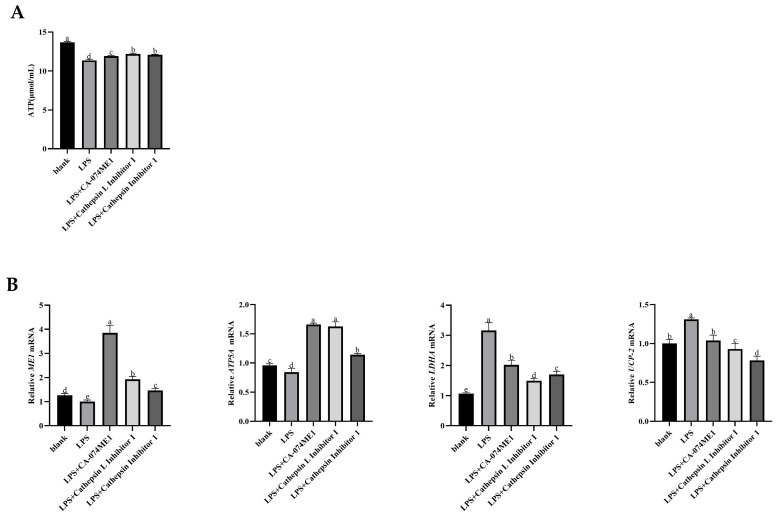
Effects of C3 activation inhibited the ATP concentration (**A**) and mRNA expressions of key molecules in ATP generation (**B**) in yak rumen epithelial cells under an inflammatory state. The values are presented as mean ± SEM (*n* = 6). Different superscripts (a–e) indicate significant differences (*p* < 0.05).

The effect of adding different a cathepsin inhibitors on ATP production in the rumen epithelial cells of yaks were shown in Figure 4A. Compared with the blank group, ATP content in rumen epithelial cells was significantly decreased in the LPS group (*p* < 0.05). After the addition of the cathepsin inhibitors, ATP content were significantly increased (*p* < 0.05).

The gene expression levels of the *ME1* gene (*p* > 0.05) and *ATP5A* (*p* < 0.05) of the ATP synthase subunit, which is involved in aerobic respiration to catalyze pyruvate to enter the tricarboxylic acid cycle, were decreased after adding LPS. However, the genes expressions were significantly increased after the addition of cathepsin inhibitor (*p* < 0.05). In the LPS group, the gene expressions of *LDHA*, a key enzyme that promotes lactic acid production after pyruvate enters anaerobic respiration, and *UCP2*, a key gene of the mitochondrial proton pump that inhibits ATP production, were significantly increased (*p* < 0.05). The expression of these genes were significantly decreased after cathepsin inhibitors were added (*p* < 0.05, Figure 4B).

### 3.5. Effects of C3a/C3aR Downstream of C3 on Pro-Inflammatory Factors of Yak Rumen Epithelial Cells Under an Inflammatory State

**Figure 5:**
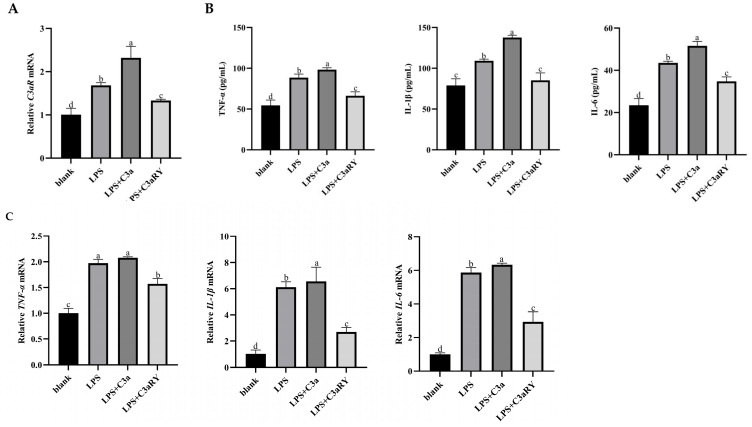
Effects of C3a/C3aR on C3aR gene expression (**A**), pro-inflammatory cytokine secretion (**B**), and related gene mRNA expression (**C**) in yak rumen epithelial cells under an inflammatory state. The values are presented as mean ± SEM (*n* = 6). Different superscripts (a–d) indicate significant differences (*p* < 0.05).

Compared with the blank group, adding LPS and LPS + C3a groups significantly increased the gene-relative expression of the *C3aR*-specific receptor (*p* < 0.05). After the addition of C3aR inhibitor C3aRY, the relative expression of *C3aR* gene decreased (*p* < 0.05, Figure 5A).

Compared with the blank group, the concentrations and gene expressions of pro-inflammatory factors *IL-6*, *IL-1β*, and *TNF-α* in rumen epithelial cells of the LPS group were significantly increased (*p* < 0.05), and the addition of C3a in LPS-induced inflammation further promoted the up-regulation of pro-inflammatory factors (*p* < 0.05). However, compared with the LPS group, the addition of C3aR inhibitor significantly decreased the content and gene expression of pro-inflammatory factors (*p* < 0.05, Figure 5B,C).

### 3.6. Effects of C3a/C3aR Downstream of C3 on ATP Production of Yak Rumen Epithelial Cells Under an Inflammatory State

**Figure 6:**
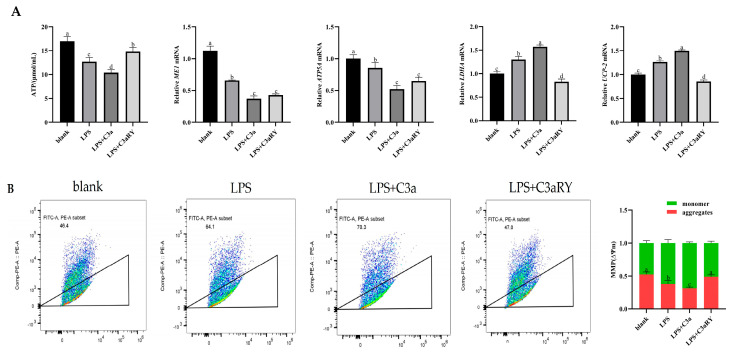
Effects of C3a/C3aR on ATP concentration, mitochondrial membrane potential, and the mRNA expression of key molecules involved in ATP generation in yak rumen epithelial cells under inflammatory conditions (**A**). In (**B**), the green represents the JC−1 monomer; the red represents the JC−1 aggregates. The values are presented as mean ± SEM (*n* = 6). Different superscripts (a–d) indicate significant differences (*p* < 0.05).

Compared with the blank group, ATP content in the LPS group and LPS + C3a group was significantly decreased (*p* < 0.05). After the addition of the LPS + C3aR inhibitor, ATP content was significantly up-regulated (*p* < 0.05). Compared with the control group, the gene expression of ME1 (*p* < 0.05) and ATP5A (*p* < 0.05) in the LPS group and LPS + C3a group was decreased, and the expression was significantly increased after the addition of C3aR inhibitor (*p* < 0.05) Moreover, the gene expressions of the key enzyme LDHA and mitochondrial proton pump UCP-2, which induce pyruvate to synthesize lactic acid, were significantly increased in the LPS group and LPS + C3a group (*p* < 0.05). After the addition, C3aRY significantly decreased the gene expression of LDHA and UP-2 (*p* < 0.05, Figure 6A).

After JC-1 staining and flow cytometry, it was found that compared with the blank group, the polymers producing red light in the LPS group and LPS + C3a group were significantly reduced, and the mitochondrial membrane potential gradually decreased (*p* < 0.05). The mitochondrial membrane potential in the LPS + C3aRY group was significantly increased compared with the LPS group (*p* < 0.05, Figure 6B).

#### Effects of C3a/C3aR Downstream of C3 on Concentrations of Tricarboxylic Acid Cycling and Anaerobic Respiratory Metabolites

**Figure 7:**
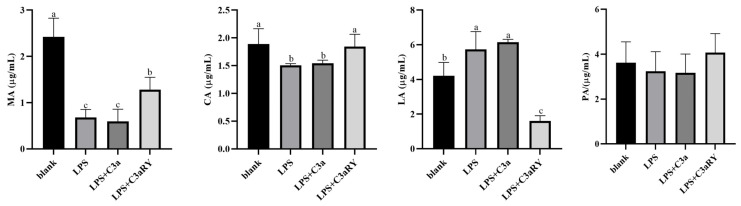
Effects of C3a/C3aR on the concentrations of metabolites in the tricarboxylic acid cycling and anaerobic respiratory. The values are presented as mean ± SEM (*n* = 6). Different superscripts (a–c) indicate significant differences (*p* < 0.05).

As the results showed in Figure 7. Compared with the blank group, the concentrations of MA and CA intermediates involved in the tricarboxylic acid cycle of aerobic respiration, in rumen epithelial cells of the LPS group and LPS + C3a group were decreased (*p* < 0.05), and the concentrations of MA and CA were increased in LPS + C3aRY group (*p* < 0.05). The concentration of anaerobic respiratory product LA in the LPS group and LPS + C3a group was increased compared with the blank group (*p* < 0.05). However, LA concentration in the LPS + C3aRY group was significantly decreased (*p* < 0.05). Compared with the blank group, the concentration of PA, a key hub of cell respiration, in the LPS group and LPS + C3a group was decreased (*p* > 0.05), and the concentration of PA in the LPS + C3aRY group was increased (*p* > 0.05), but there was no significant difference.

### 3.7. Effects of C3a/C3aR Downstream of C3 on cAMP/PKA Pathway Related Gene Expression

**Figure 8:**
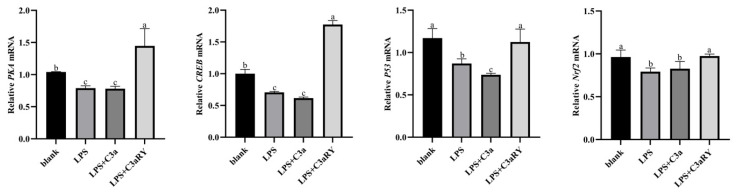
Effects of C3a/C3aR on the mRNA expressions of cAMP/PKA pathway. The values are presented as mean ± SEM (*n* = 6). Different superscripts (a–c) indicate significant differences (*p* < 0.05).

As the results showed in Figure 8. The gene expressions of *PKA* and *CREB*, two key genes of the cAMP/PKA pathway, were significantly decreased in the LPS group and LPS + C3a group compared with the blank group (*p* < 0.05). However, the gene expressions were significantly increased after the addition of C3aR inhibitor (*p* < 0.05). The gene expression levels of *P53* and *Nrf2*, two downstream factors related to the cAMP/PKA pathway, were decreased in the LPS group and LPS + C3a group compared with the blank group (*p* < 0.05). The gene expressions in the LPS + C3aRY group were significantly increased compared with the LPS group (*p* < 0.05).

### 3.8. Effects of C3a/C3aR Downstream of C3 on Expression of Tight Junction Protein Gene

**Figure 9:**
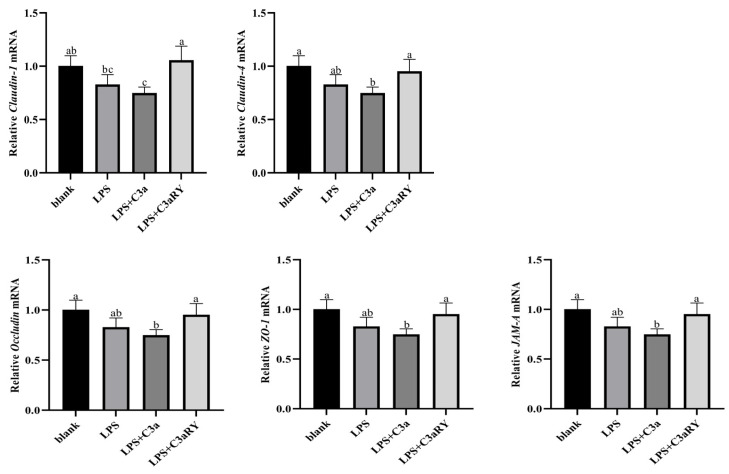
Effects of C3a/C3aR on the genes expressions of tight junction protein. The values are presented as mean ± SEM (*n* = 6). Different superscripts (a–c) indicate significant differences (*p* < 0.05).

As the results showed in Figure 9. Compared with the blank group, the relative gene expression of tight junction proteins *Claudin-1*, *Claudin-4*, *Occludin*, *ZO-1*, and *JAM-A* in the LPS + C3a group significantly decreased (*p* < 0.05). The relative gene expression of tight junction proteins *Occludin*, *Claudin-4*, *ZO-1*, and *JAM-A* in the LPS + C3aRY group was increased but not significantly compared with the LPS group (*p* > 0.05), except for Claudin-1 (*p* < 0.05).

## 4. Discussion

Previous studies have shown that the increase in LPS concentration in the rumen of growths retardation yaks can up-regulate pro-inflammatory factors such as IL-1β and TNF-α, leading to immunoinflammatory reaction in rumen epithelium, damages rumen epithelial barrier function, and inhibits feed conversion and efficient growth of yaks [17,27]. Moreover, previous proteomics research revealed that the rumen epithelial cells of growth retardation yak experienced hindrances in ATP synthesis, and the concurrent ATP concentration exhibited a positive correlation with daily weight gain [2]. Meanwhile, studies have shown that in immune cells, the activation of C3 affects intracellular ATP synthesis and energy metabolism. However, for rumen epithelial cells, non-immune cells, the effect on ATP synthesis after the intracellular activation of C3 has not been elaborated.

### 4.1. LPS-Induced Inflammation Model and Intracellular Activation of C3 in Rumen Epithelial Cells of Yak

Previous studies have indicated that when the LPS concentration increases from 0 to 200,000 EU/mL (20 µg/mL), there is a significant suppression of rumen epithelial cell activity, along with an increase in pro-inflammatory factor levels, thus establishing a cellular inflammation model [17,28,29]. This study induced the secretion of pro-inflammatory factors such as TNF-α, IL-1β, and IL-6 in yak rumen epithelial cells by adding different concentrations of LPS. It was found that at an LPS concentration of 20 µg/mL, an inflammation model of rumen epithelial cells was successfully established for this experiment. Additionally, some studies have found that LPS can activate C3 to produce C3a and C3b, and the activation of C3 is involved in inhibiting intracellular ATP production [10,30]. This study found that the addition of exogenous LPS led to an increase in the production of C3a and C3b in rumen epithelial cells, suggested the activation of immune response by treating with 20 µg/mL LPS, while ATP production decreased.

### 4.2. Effects of Inhibition of Intracellular C3 Activation on ATP Production in Rumen Epithelial Cells of Yak Under Inflammatory Conditions

In this study, to determine whether the activation products of C3 in rumen epithelial cells are related to the inhibition of ATP production, protease inhibitors were added to rumen epithelial cells under LPS-induced inflammatory conditions. Under LPS-induced inflammatory conditions, the addition of cathepsin inhibitors reduced the activation products of C3 (C3a and C3b), downregulated the expression of *C3* and *C3aR*, decreased the secretion of inflammatory factors, and significantly increased ATP production. In addition to investigating the effect of intracellular C3 activation on the mechanism of ATP production in rumen epithelial cells, this experiment measured the gene expression of *ME1*, *ATP5A*, *LDHA*, and *UCP2*. ME1 functions as a key enzyme connecting the TCA cycle and NADPH generation, supporting antioxidative defense and lipid metabolism [31]. ATP5A represents a marker for mitochondrial oxidative phosphorylation, whereas LDHA reflects a shift toward anaerobic glycolysis [32], and UCP2 functions as an uncoupling protein that decreases mitochondrial ATP efficiency through proton leakage [33]. The gene expression levels of *ME1* and *ATP5A* were upregulated, and *LDHA* and *UCP2* were downregulated after cathepsin inhibitors were added. These results suggest that inhibiting intracellular C3 activation in rumen epithelial cells by adding cathepsin inhibitors may be associated with improved mitochondrial function and enhanced ATP production, potentially involving the aerobic respiration pathway, although further studies are needed to confirm this mechanism.

### 4.3. Effect of C3a/C3aR Downstream of C3 on Pro-Inflammatory Factors and ATP Production of Yak Rumen Epithelial Cells Under an Inflammatory State

In this study, it was found that the expression of C3 in rumen epithelial cells could be activated after the addition of LPS. According to previous studies, the intracellular activation of C3 is the core of immune defense and immune regulation, and the most critical step is the production of C3 into C3a and C3b [34]. In the immune cells of mammals, C3a is considered an allergic toxin. When C3a binds to its specific receptor C3aR, it inhibits mitochondrial respiration and ATP production, thereby affecting energy generation [35,36]. This study found that exogenous addition of C3a in rumen epithelial cells also increased the concentration of pro-inflammatory factors and decreased the content of ATP. This phenomenon can be explained by the dual effects of the C3a–C3aR signaling pathway. On the one hand, the binding of C3a to its receptor C3aR activates downstream inflammatory signaling cascades, such as NF-κB and MAPK, thereby enhancing the production of pro-inflammatory factors including IL-6, TNF-α, and IL-1β [8]. On the other hand, it has been reported that the interaction between C3a and C3aR impairs mitochondrial respiration, which consequently restricts oxidative phosphorylation and reduces ATP generation [37]. Moreover, Previous studies have shown that feeding mice a C3aR inhibitor to block the binding of C3a to its receptor can alleviate inflammation and metabolic disorders in obese mice [21]. In this study, it was found that after adding the C3aR inhibitor C3aRY in vitro, there was a decrease in the concentration of pro-inflammatory factors in the rumen epithelial cells and an increase in ATP production. This suggests that blocking the interaction between C3a and C3aR can alleviate inflammatory signaling, reduce excessive energy consumption associated with immune activation, and restore mitochondrial respiratory efficiency. These results indicate that the decrease in ATP production in yak rumen epithelial cells under inflammatory conditions is related to the binding of the C3 activation product C3a to its downstream receptor C3aR. This interaction can be inhibited by adding a C3aR inhibitor, which upregulates ATP production.

In addition, studies have found that organic acids such as citric acid (CA), malic acid (MA), and pyruvic acid (PA) are important components of the tricarboxylic acid (TCA) cycle. Through the TCA cycle, ATP is produced as energy, which enhances the digestibility of certain nutrients and thus participates in the metabolic pathways of the organism [38]. However, lactic acid, as a product of anaerobic respiration, yields relatively little ATP during the anaerobic respiration process [39]. In this study found that the ATP generation from the aerobic respiration were depressed in the rumen epithelial cells under inflammatory conditions, and adding the inhibitor C3aRY to rumen epithelial cells under inflammatory conditions increased the concentrations of tricarboxylic acid cycle metabolites MA, CA, and PA, while decreasing the concentration of the anaerobic respiration product lactic acid (LA). These results suggest that the effect of C3 on ATP production in yak rumen epithelial cells may be regulated by the binding of its product C3a to the receptor C3aR. Inhibiting the binding of C3a to C3aR can upregulate ATP production, possibly by promoting the tricarboxylic acid cycle and reducing anaerobic respiration. These results suggested that supplementing energy generating substrates for the gastrointestinal cells, such as glutamine and butyrate, had the potential role to promote gastrointestinal tract health and body growth under inflammatory conditions.

### 4.4. Effects of C3a/C3aR Downstream of C3 on cAMP/PKA Pathway-Related Gene Expression of Yak Rumen Epithelial Cells Under an Inflammatory State

Cyclic adenosine monophosphate (cAMP) is one of the earliest discovered second messengers, primarily regulating the transcription of multiple target genes through protein kinase A (PKA) and its downstream effectors, such as cAMP response element-binding protein (CREB). It plays a crucial role in intracellular signal transduction [40]. Previous studies have found that the cAMP/CREB pathway can enhance cellular immune function and energy metabolism by activating the signaling molecules *Nrf2* and *P53* [41,42]. Additionally, some studies have found that C3aR, as a G protein-coupled receptor, is closely related to the cAMP/PKA signaling pathway [43]. In this study, it found that after the addition of C3aR inhibitors in rumen epithelial cells under inflammatory conditions, the expression of related genes of cAMP/PKA signaling pathway and the expression of genes of *Nrf2* and *P53* were increased. This indicates that in the inflammatory state of rumen epithelial cells, inhibiting the binding of C3a and C3aR can upregulate the expression of downstream factors *PKA*, *CREB*, *P53*, and *Nrf2* in the cAMP/PKA signaling pathway, thereby maintaining normal cellular physiological functions and ATP production. Previous studies have reported the energy-conserving roles of C3a and its receptor in the adipocyte. The antagonist of C3aR also reduced pro-inflammatory factors in adipose tissue [21]. The C3a promoted energy conservation and inhibited lipolysis potentially through inhibiting cAMP signaling. The intracellular cAMP is produced from ATP by adenylate cyclase activity, suggesting a potential connection between C3a and ATP production. Asgari et al., 2013 found that C3a regulated the ATP efflux in human monocytes [35]. These studies demonstrated that C3a regulated ATP generation potentially through acting on cAMP signaling pathway, but the mechanisms of cAMP signaling regulating on ATP generation need further research.

### 4.5. Effect of C3a/C3aR Downstream of C3 on Expression of Tight Junction Protein Gene

Tight junction proteins are in the upper layer of epithelial cells, which play an important role in preventing harmful substances, such as ruminal LPS, from entering the submucosal layer through the epithelial intercellular spaces and regulating epithelial permeability and nutrient transport [44]. Previous studies have found that *Occludin* protein expression in the jejunal epithelium of slow-growing piglets is lower than that of normal piglets [45]. Some studies have found that protein expression and barrier function of tight junction proteins *ZO-1*, *Occludin*, and *Claudin-1* in the rumen and jejunum of growth-retarded yaks are inhibited [46]. Whether these inhibitory effects are related to the active expression of C3 deserves further research. The experiment found that the exogenous addition of C3a and C3aRY in the inflammatory state of yak rumen epithelial cells revealed that the addition of C3aRY, by inhibiting the binding of C3a to C3aR, could modulate the expression of tight junction proteins, ensuring the integrity of the rumen epithelium and the transport of nutrients, thereby normalizing ATP production.

## 5. Conclusions

The results of this study demonstrated that intracellular C3 activation inhibited ATP production in yak rumen epithelial cells under an inflammatory response. Specifically, LPS stimulation triggered the generation of C3a, which binds to its receptor C3aR, leading to mitochondrial dysfunction characterized by decreased membrane potential. This interaction suppressed aerobic respiration, as evidenced by the downregulation of tricarboxylic acid cycle metabolites and *ATP5A* gene expression. This process may be regulated by the cAMP/PKA signaling pathway. Future research should focus on in vivo validation of strategies targeting the C3a-C3aR axis and explore specific regulatory elements within the C3 signaling pathway to develop practical interventions aimed at improving rumen health and growth performance in yaks.

## Data Availability

The data presented in this study is available on request from the authors.

## Supplementary Materials

The following supporting information can be downloaded at https://www.mdpi.com/article/10.3390/vetsci12090841/s1, Table S1: Primer sequences for Real-time PCR.
