# Supplementary material for: LPS-Induced Intracellular Complement 3 Activation Regulated ATP Production in Yak Rumen Epithelial Cells
Source: Vet Sci. 2025 Aug 31;12(9):841. doi: 10.3390/vetsci12090841 (PMC12474471; doi:10.3390/vetsci12090841)
Supplement: Supplementary file 1 [file vetsci-12-00841-s001.zip › vetsci-3830268-supplementary.pdf]

Table S1 Primer sequences for Real-time PCR

Gene	Accessin No.[1]	Primes sequence (5′-3′)[2]	Size (bp)
β-Actin	XM_005897464.1	F:TGGCGCTTGACTCAGGATTT<br>R:CAATCAAGTCCTCGGCCACA	102
TNF-α	XM_005904178.1	F:TGAAGGAAGAGGAGAGGCTCATCG<br>R:GTGGTCATCGGAGTTGCTGGTG	107
IL-6	XM_005901249.2	F:CACTGACCTGCTGGAGAAGATGC<br>R:CCGAATAGCTCTCAGGCTGAACTG	115
IL-1β	NM_174093.1	F:CTCCGACGAGTTTCTGTGTGACG<br>R:GAGAGGAGGTGGAGAGCCTTCAG	120
C3	XM_005890706.2	F:CAACCTCATCGCCATAGACTCCAAG<br>R:GATTCCATCAGGCTTCTGCTTCTCC	82
ME1	XM_005903318.2	F:TGATCCCGTTACCCTTCCGAGTG<br>R:CTCAGTCCACACGCCACAACA	105
LDHA	XM_014479751.1	F:TTCAGCTCGCTTCCGTTATCTCATG<br>R:ACACCAGCAACATTCACTCCACTC	129
CTSB	XM_005886688.2	F:CAACGAGAAGGAGATCATGGCAGAG<br>R:TAGCAGGAAGTCCGAGTACACAGAG	82
CTSL	NM_001083686.2	F:GGAGAAGGCTCTTATGAAGGCAGTG<br>R:AGAACAGCATGATCCACGGATTCAC	144
ATP5A	XM_005892346.2	F:GCCCAGCAAGATCACAAAATTT<br>R:GGGCCTGATGTTGGCTGATA	65
UCP-2	XM_005893537.2	F:CCCAATGTCGCTCGCAAT<br>R:AGTGTCCTTGATGAGGTCGTAGGT	210
Nrf2	NM_001011678.2	F:ATGACAAGCTGGCTGAGACT<br>R:GTTCACTGTCAACTGGCTGG	102
P53	XM_005894802.2	F:TAACCTTCTGTCCTCCGAGC<br>R:TCTGGGAGGGGACAAATGAC	107
CREB	XM_014479973.1	F:ATGAAGCAGCAGTCATGGGGT	182

		R:GAATGTGGAGGCTGGGACT	
PKA	XM_005887423.2	F:AGACCTCGTAGACTTCGCG<br>R:ACTGACACTCGCCGATCAA	207
Occludin	XM_005889348.2	F:GCCTGTGTTGCCTCCACTCTTG<br>R:CCATAGCCATAACCGTAGCCATAGC	143
Claudin-1	XM_005897671.2	F:CCCGTGCCTTGATGGTGATTGG<br>R:CATCTTCTGTGCCTCGTCGTCTTC	110
ZO-1	XM_014476599.1	F:GCATGATGATCGTCTGTCCTACCTG<br>R:CCGCCTTCTGTGTCTGTGTCTTC	108
Claudin-4	XM_005892850.2	F:CAGCAGCGAGTCGTACACCTTG<br>R:TCATCGGCAGCAACATCGTCAC	110
JAM-A	XM_005909189.2	F:GTGCCTCCATCCAAGCCTACAATC<br>R:GGCATCTCTACTCCATCCTTGAACC	134

[1] National Center for Biotechnology Information (NCBI; Bethesda, MD).

[2] F = forward; R = reverse.
